# Early detection of pulmonary exacerbations in children with Cystic Fibrosis by electronic home monitoring of symptoms and lung function

**DOI:** 10.1038/s41598-017-10945-3

**Published:** 2017-09-27

**Authors:** Marieke van Horck, Bjorn Winkens, Geertjan Wesseling, Dillys van Vliet, Kim van de Kant, Sanne Vaassen, Karin de Winter-de Groot, Ilja de Vreede, Quirijn Jöbsis, Edward Dompeling

**Affiliations:** 1 Department of Paediatric Respiratory Medicine, School for Public Health and Primary Health Care (CAPHRI), Maastricht University Medical Centre (MUMC+), Maastricht, The Netherlands; 2 Department of Methodology and Statistics, CAPHRI, MUMC+, Maastricht, The Netherlands; 3 Department of Respiratory Medicine, CAPHRI, MUMC+, Maastricht, The Netherlands; 4 Department of Paediatric Respiratory Medicine, Wilhelmina Children’s Hospital, University Medical Centre Utrecht (UMCU), Utrecht, The Netherlands; 5 Department of Paediatric Respiratory Medicine, Leiden University Medical Centre (LUMC), Leiden, The Netherlands

## Abstract

Pulmonary exacerbations (PEx) in Cystic Fibrosis (CF) are associated with an increased morbidity and even mortality. We investigated whether early detection of PEx in children with CF is possible by electronic home monitoring of symptoms and lung function. During this one-year prospective multi-centre study, 49 children with CF were asked to use a home monitor three times a week. Measurements consisted of a respiratory symptom questionnaire and assessment of Forced Expiratory Volume in one second (FEV1). Linear mixed-effects and multiple logistic regression analyses were used. In the 2 weeks before a PEx, the Respiratory Symptom Score (RSS) of the home monitor increased (p = 0.051). The FEV1 as percentage of predicted (FEV1%pred) did not deteriorate in the 4 weeks before a PEx. Nevertheless, the FEV1%pred at the start of exacerbation was significantly lower than the FEV1%pred in the non-exacerbation group (mean difference 16.3%, p = 0.012). The combination of FEV1%pred and RSS had a sensitivity to predict an exacerbation of 92.9% (CI 75.0–98.8%) and a specificity of 88.9% (CI 50.7–99.4%). The combination of home monitor FEV1%pred and RSS can be helpful to predict a PEx in children with CF at an early stage.

## Introduction

Pulmonary exacerbations in Cystic Fibrosis (CF) are important events, associated with an accelerated decline in lung function and an increased morbidity and even mortality^1^. Prevention of pulmonary exacerbations (PEx) is one of the main goals in the management of CF^2^. Currently, treatment of a PEx starts when a patient presents with an increase in (respiratory) complaints, a deterioration in lung function and/or weight loss. It often takes some time before a patient with more complaints seeks medical care. This patient delay may introduce a subsequent delay in starting antibiotic treatment, potentially resulting in a more severe course of the PEx, and a higher risk of permanent lung function loss.

There are some known risk factors for PEx in children, including frequency of previous PEx, a lower baseline Forced Expiratory Volume in one second (FEV_1_), and female sex^3^. However, in individual cases these factors do not help to decide when to start therapy timely. As PEx cannot be predicted reliably yet, there is a need for a method to detect PEx at an earlier stage. Electronic home monitoring may be useful to detect changes in lung function and respiratory complaints at an early stage. It provides the opportunity to assess symptoms and lung function on a daily or weekly basis. Data can be sent to the CF centre where they can be evaluated. In this way, disease deterioration and the occurrence of PEx may be detected earlier.

Data of electronic home monitoring in CF to detect exacerbations are scarce^4^. A small study in adults with CF assessed whether early detection of PEx by home monitoring was possible^4^. In children with asthma, we recently demonstrated that home monitoring is better able to detect cases with less well-controlled disease than a validated questionnaire during hospital visits^5^.

The aim of the present study was to test the hypothesis that electronic home monitoring of respiratory symptoms and lung function is a valuable tool to detect PEx in children with CF at an early stage.

## Methods

### Study design and population

Children with CF aged 5 to 19 years were included in this one year, multicentre, observational cohort study (clinicaltrial.gov NCT01241890). Children were recruited from three CF centres in the Netherlands (Maastricht, Utrecht and Amsterdam).

CF was defined as the presence of characteristic clinical features (persistent pulmonary symptoms, meconium ileus, failure to thrive, steatorrhea) in combination with an abnormal sweat test (chloride >60 mM) and/or two CF mutations^6^. Exclusion criteria were: (1) severe cardiac abnormalities; (2) mental disability; (3) no technically adequate performance of measurements (especially related to lung function assessments); (4) on waiting list for lung transplantation; (5) children colonized with *Burkholderia cepacia* or *Methicillin Resistant Staphylococcus aureus*; (6) participation in an intervention trial.

Ethical approval was obtained from the Medical Ethical Committee of the Maastricht University Medical Centre. Informed consent was signed by all parents, and by children aged 12 years and over. All methods were performed in accordance with the relevant guidelines and regulations.

### Study parameters

For the period of one year, we surveyed the children by means of regular clinical visits every two months and thrice-weekly home monitoring of lung function and symptoms. Demographic information and medical history were collected at inclusion. During each clinical visit, changes in medication were reported and spirometry was performed. During the one-year follow up, all PEx were recorded.

### Home monitoring

All children received a handheld Jaeger home monitor (AM 2+, CareFusion, Houten, The Netherlands). A prototype of this monitor was tested for accuracy, reproducibility and interdevice variability and appeared to meet the American Thoracic Society (ATS) standards for monitoring devices^7^.

Children were asked to use the home monitor three times a week at the same time of the day. By using the home monitor, children recorded the presence and severity of respiratory symptoms (cough, sputum and dyspnoea) digitally by answering three questions. The severity of symptoms was scored on a range from 0 (no complaints) to 3 (a lot of complaints). The respiratory symptom score (RSS) was defined as the sum of these three questions (range 0 to 9) and was used for the analysis. Subsequently, lung function measurements consisted of three forced vital capacity (FVC) manoeuvres with maximal effort. All children and parents were thoroughly instructed how to perform a correct FVC manoeuver. The mean FEV_1_ as % of predicted (FEV_1_%pred) of each day was calculated and used for the analysis.

RSS and lung function measurements were stored in the device. The families were asked to transfer the data into a secured web-based portal (Avetana, GmbH, Hoechberg, Germany) once a week. Families who did not send their data weekly, received a reminder to increase adherence.

Adherence to the home monitor was defined as completion of 70% of the maximum number of requested home monitor measurements over one year.

### Primary outcome: detection of PEx

The primary outcome was the detection of a PEx, which was defined in two ways: first according to the definition used in the Early Pseudomonas Infection Control (EPIC) trial^8^. The presence of a PEx was established by one of the major criteria alone, or two of the minor criteria, and fulfilment of symptom duration (duration of sign/symptoms ≥5 days or significant symptom severity) (s*ee* Table 1 *supplement*). Second, when the responsible paediatric pulmonologist started a course of therapeutic antibiotics considering the clinical symptoms as an expression of a PEx.

**Table 1:** Baseline characteristics of study cohort.

Characteristic	Total cohort (n = 49)
Age, mean (SD)	10.3 (3.6)
Male sex, N (%)	31 (63)
Homozygous dF508, N (%)	36 (74)
PEx in 2 years before inclusion, N (%)	23 (47)
*Pseudomonas aeruginosa* at inclusion*, N (%)	15(31)
ABPA at inclusion*, N (%)	2 (4)
BMI, median (IQR)	16.8 (16.0–18.1)
BMI-SDS, mean (SD)	0.14 (0.8)
FEV_1%_ predicted value, mean (SD)	87.4 (18.1)
FVC % predicted value, mean (SD)	92.4 (16.4)
FEV_1_/FVC, mean (SD)	0.8 (0.1)
RV % predicted value, mean (SD)^#^	130.9 (42.7)
TLC % predicted value, mean (SD)^#^	101.1 (12.0)
Prophylactic antibiotics, N (%)	28 (57)
Inhalation corticosteroids, N (%)	16 (33)

*Treated because of positive sputum culture. ^#^Total n = 37 (12 children did not perform static lung function). Abbreviations: ABPA, Allergic Bronchopulmonary Aspergillosis; BMI, body mass index; FEV_1_, Forced Expiratory Volume in 1 second; FVC, Forced Vital Capacity; PEx, pulmonary exacerbation; RV, Residual Volume; SD, Standard deviation; TLC, Total Lung Capacity. All p > 0.05.

PEx were treated according to the Dutch Central Guidance Committee (CBO) guideline^9^. which resembles European^6^ and American CF guidelines^10^.

### Statistical Analysis

Baseline characteristics were expressed as mean (standard deviation [SD]) or median (interquartile range [IQR]) where appropriate for numerical variables, and as number (percentage) for categorical variables.

We used the first PEx for the longitudinal analysis of FEV_1_%pred and RSS from four weeks before until the start of a PEx. If this PEx was within four weeks after inclusion, we used the subsequent PEx as long as the time between the end of the antibiotic course for the previous PEx and the start of the subsequent PEx was again at least four weeks.

A linear mixed-effects model was used to test differences in home monitor data during four weeks prior to the selected PEx between patients with a PEx (exacerbation group) and patients without any PEx (no exacerbation group). The median number of days before the PEx in the exacerbation group was used as reference point (t = 0) for the control group, measurements up to four weeks before this time point were used in the analysis. Group (exacerbation or no exacerbation), time to PEx in weeks (t = −4 to t = 0), and time*group were included as fixed factors. Other fixed factors were: centre, gender, age at inclusion (in years), *Pseudomonas Aeruginosa* at inclusion, PEx in the two years before inclusion, and season, as these factors were expected to be related to PEx. For the random part of the model, different options were considered, i.e. only random intercept, or random intercept and slope (variance components or unstructured). The option with the smallest Akaike’s information criterion (AIC) was selected^11^. Sensitivity/robustness analyses were performed, in which the influence of the used definition of a PEx, inclusion of only the first PEx during the study, or changing the period before a PEx from four to eight weeks, on the results was checked.

In addition, multiple logistic regression analysis was used to study the effect of FEV_1_%pred and RSS from four weeks to one week before a PEx (or reference point in case of no PEx) on probability of obtaining a PEx. To be of predictive use, the week just before a PEx was excluded. The area under the ROC curve was used as a measure of prediction quality. As for optimal cut-off point of predicted probability, the Youden index was used, which maximizes the sum of sensitivity and specificity^12^. An internal validation was performed using a bootstrapping method (1000 bootstrapping samples), where the optimism in predictive quality of the model (AUC) due to overfitting was computed and adjusted for.

Data were analysed with IBM SPSS Statistics for Windows (version 22.0. Armonk, NY), while we used the R package entitled ‘rms’ for the internal validation of the prediction model (version 3.1-0. Vienna, Austria).

## Results

### Patient Characteristics

Forty-nine children (mean age 10.3 years) participated in this study. Patient characteristics are shown in Table 1.

**Figure 1:**
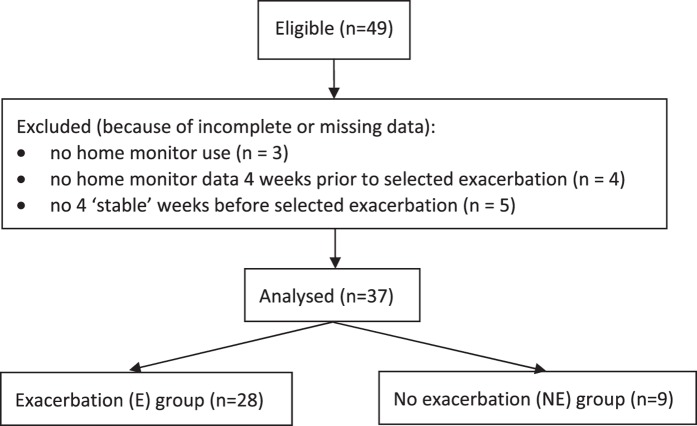
Flowchart of included study population.

As shown in Fig. 1, three patients did not use the home monitor, two due to technical reasons, and one child dropped out before using the home monitor. Nine children were excluded from the analysis as no four weeks of home monitor data before a PEx were available in these children (e.g. due to an PEx within four weeks or because of missing data). Therefore, data of 37 patients could be analysed. There were no clinically relevant or statistically significant differences in baseline characteristics between children included or excluded from the analyses or between the exacerbation and the control group.

### Course of the FEV_1_%pred and RSS in exacerbation (E) and no exacerbation (NE) groups

Twenty-eight children participated in the E group: data on the first and the subsequent PEx were used in 18 and 10 children respectively. The median time from inclusion to the selected PEx was 86 days. The NE group consisted of nine children who did not experience a PEx during the one-year study period.

**Figure 2:**
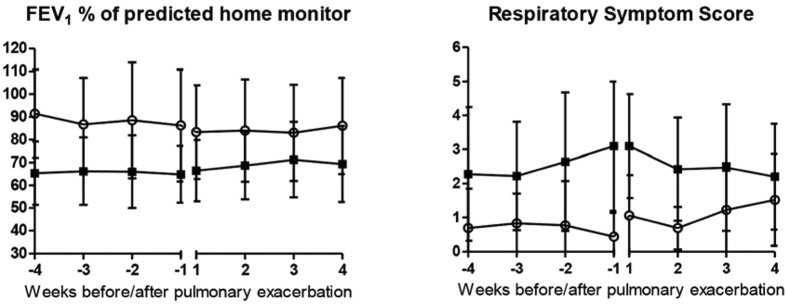
Course of the estimated mean (**A**) FEV_1_%pred or (**B**) Respiratory Symptom Score 4 weeks before and after a pulmonary exacerbation for the E group (n = 28; filled squares) and NE group (n = 9; open circles). Bars represent corresponding SEs.

In Fig. 2A and B, the course of the estimated mean FEV_1_%pred and estimated mean RSS in the four weeks before and after the onset of the PEx for both the E group and the NE group are demonstrated.

### Difference in FEV_1_%pred and RSS between exacerbation and no exacerbation group

In Table 2, the results of the mixed-effects model analysis are shown. The time trend for the FEV_1_%pred in the 4 weeks before exacerbation was not significant (p = 0.650), although the FEV_1_%pred at the start of the PEx (T = 0)was significantly lower in the E group compared to the control group (mean difference 16.3%, p = 0.012). The RSS showed a trend towards a significant increase in the weeks before exacerbation (p = 0.051). As the FEV_1_%pred, the RSS at the start of the PEx was significantly worse in the E group compared to the NE group (mean difference 2.97 points on a scale of 0 to 9, p < 0.001).

**Table 2:** Estimated means and difference in estimated means (at t = 0 and time trend) between exacerbation and control group based on linear mixed-effects model^a^.

		Estimated means (SE)			Estimated mean difference (95%CI)			
		T = −4	T = −2	T = 0	T = 0	p	Time trend	p
FEV_1_%pred	E group	69.33 (11.52)	68.58 (11.37)	67.82 (11.30)	−16.30 (−28.75, −3.85)	0.012	0.067 (−0.232, 0.366)	0.650
	NE group	87.51 (12.61)	85.81 (12.25)	84.12 (12.15)				
RSS	E group	2.25 (1.38)	2.76 (1.35)	3.27 (1.33)	2.97 (1.52, 4.42)	<0.001	0.051 (0.000, 0.102)	0.051
	NE group	0.70 (1.54)	0.50 (1.46)	0.30 (1.43)				

Abbreviations: E, exacerbation; NE, no exacerbation; FEV_1%_ predicted, forced expiratory volume in 1 second percentage of predicted; RSS, Respiratory Symptom Score. ^a^Reference categories: site University Medical Centre Utrecht, female sex, age 10.3 years, exacerbations in 2 years before inclusion, Pseudomonas Aeruginosa at inclusion, season autumn.

Sensitivity/robustness analyses showed similar results when only PEx according to the EPIC trial definition were used, when only the first PEx during the study were included, or when the time period before a PEx was increased to eight weeks.

### Prediction model of PEx

From the above mentioned data and analysis, it was evident that a lower FEV_1_%pred and a higher RSS preceded a PEx. Therefore, these parameters were used as independent variables in a multiple logistic regression analysis in order to study the effect on the probability of obtaining a PEx. We used the mean values over the period from four weeks up to one week before a PEx. Both the mean FEV_1_%pred and the mean RSS appeared to be independent predictors of PEx and the combination resulted in an area under the ROC curve of 0.87.

**Figure 3:**
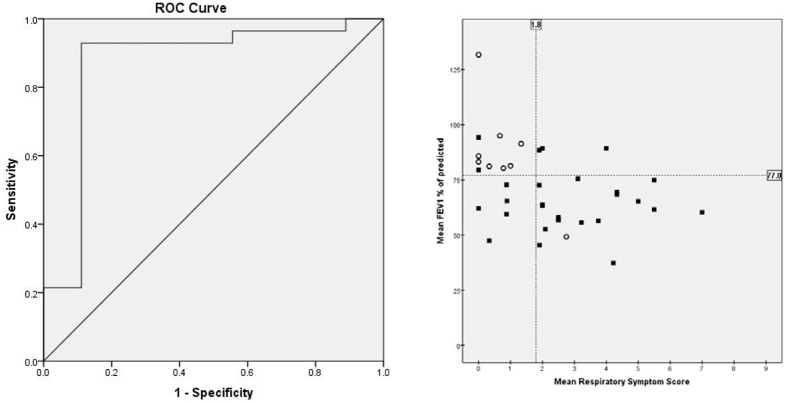
(**A**) ROC curve of predictive model of pulmonary exacerbations and (**B**) combination threshold of mean FEV_1_%pred and mean RSS to predict a PEx. Filled squares correspond to children in the exacerbation group, open circles correspond to children in the no exacerbation group.

The optimum cut-off point, i.e. the one which maximizes the sum of sensitivity and specificity (Youden Index), gave a sensitivity of 92.9% (CI 75.0–98.8%) and a specificity of 88.9% (CI 50.7–99.4%) for the combination of the mean FEV_1_%pred and the mean RSS of the home monitor. A combination threshold of mean FEV_1_%pred lower than 77% and mean RSS higher than 1.8 was predictive for a PEx (Fig. 3B).

### Adherence to the home monitor

Fifty-four % of the children demonstrated good adherence to the home monitor. The median percentage of completed data compared to the total amount of requested data of all children was 77% (IQR 45–88%).

## Discussion

This study in children with CF showed an increase in the RSS from the home monitor, especially in the 2 weeks before exacerbation. Moreover, the RSS was already worse 4 weeks before a PEx in the E group compared to the NE group. In contrast, no deterioration in FEV_1_%pred in the 4 weeks before a PEX was found, although the FEV_1_%pred at the start of the exacerbation was lower in the exacerbation group compared to the NE group. The combination of the mean FEV_1_%pred and mean RSS of four weeks to one week before a PEx resulted in good sensitivity and specificity to predict an exacerbation in our study. To improve long term prognosis of children with CF, early detection of PEx is essential. Electronic home monitoring of symptoms and lung function seems a promising tool to detect PEx in children with CF at an early stage. However, a validation study in a larger cohort should be performed.

While the FEV_1_%pred and the RSS differed significantly between the E group and the NE group, no further decrease in FEV_1_%pred was found in the weeks or days preceding a PEx. In clinical practice physicians sometimes notice an acute decrease in lung function but more often there appears to be a slow decrease during weeks or even months when patients present themselves with increased symptoms. Lung function deterioration in CF is related to ongoing chronic airway inflammation, thick and sticky mucus with plugging and atelectasis, injury to the airways with bronchiectasis and fibrosis, which is triggered by viral infections, bacterial overgrowth, and bacterial infections/exacerbations^13^. Although symptoms were already worse in the four weeks preceding the exacerbation, further deterioration was observed in the two weeks before the PEx. In this way, symptoms seem to be a more sensitive and acute indicator of a PEx than lung function. This is in accordance with exacerbations in patients with COPD, where increased symptoms and a patient’s reported diagnosis are more important than lung function^14^.

Adherence to the home monitor could be further optimised: 77% of the requested data were really gathered by the children and the parents, and 54% of the children had good to optimal adherence (according to the current definition of assessing at least assessing 70% of the data). So far, we are not informed about the minimal or the optimal monitor frequency. It may be that with 77% of the requested monitor data, still a valuable monitoring and prediction of exacerbations is possible. An explanation for the missing data may be the high burden of disease which children with CF already experience. Another reason which may have lowered adherence might be that, despite all efforts, transfer of home monitor data to the online portal was not flawless and several families needed support. In this study, home monitor data had not consequence for treatment. We expect that the adherence will further increase when the monitor data have real impact on disease management and will be used to prevent exacerbations. In this case, children and parents will be more motivated to use the monitor. In the present study, the home monitor was used three times a week. It may be that the adherence is better when the home monitor is used on a daily basis as part of the daily routine of patients. The current relative low price of the home monitors (75 to 200 euros) increases the chance on a cost-effective eHealth intervention with the monitor, as has been suggested by a recent economic study^15^.

Our findings are in line with a previous study of Sarfaraz *et al*. in adolescents with CF^4^. In this study, fifty-one children were included to use a home monitor for six months. Only 19 patients completed recordings that could be evaluated. Nevertheless, Sarfaraz *et al*. found that daily home monitoring contributed to the early detection of PEx, which subsequently may have lowered the need for IV antibiotics^4^. Lechtzin *et al*. conducted a randomised non-blinded multicentre trial in 320 CF patients (from 14 years) comparing usual care to home monitoring of spirometry and respiratory symptoms using the AM 2+ home monitor twice a week during one year^16^. However, the results of this trial have not been published yet. Grzincich G. *et al*. conducted a pilot trial of a home telemonitoring system with spirometry and oxygen saturation involving 30 randomly selected adults with CF, showing that telemonitoring was useful in assessing the patients’ health status and in decreasing the CF centre workload^17^. An economic analysis of home telemonitoring in CF patients for a follow-up period of 10 years was performed by Tagliente I. and coworkers. The comparison between the standard clinical care and the clinical trial with telemonitoring showed a potential saving by telemonitoring of €40,397.00 per patient for a 10 year period^15^.

In asthma, home monitoring is more extensively studied, although only a few studies investigated the effects of home monitoring of the combination of lung function and respiratory symptoms^18^. In a recent study of our research group in 96 children with asthma, more cases of less well-controlled asthma were detected by prospective home monitoring of FEV_1_ and symptoms than by a validated questionnaire during hospital visits^5^. This demonstrates the potential of home monitoring in chronic respiratory diseases in children.

The strengths of our study are: (1) the longitudinal study design with the extended follow-up of 1 year; (2) frequent, thrice-weekly home monitor assessments; (3) home monitor measurements combining lung function and respiratory symptoms and (4) the use of two definitions of a PEx, limiting potential misclassification of children.

A limitation may be that although the RSS was based on previous research^19^. it is not validated and internationally acknowledged yet. Furthermore, the small sample size of our study should be mentioned. External validation of our findings in larger cohort should be performed.

The clinical implication of our study is that electronic home monitoring of symptoms and FEV_1_ can probably be used for the early detection of PEx. In combination with early antibiotic treatment, it may be possible to prevent (part of) the exacerbations in CF. This probably will have a beneficial influence on quality of life, lung function, and perhaps even prognosis. A new study in a larger cohort is necessary to confirm that early detection and improving outcomes are possible. In summary, prevention of PEx is important but in order to prevent PEx we first have to detect these events at an early stage. In the 2 weeks before an exacerbation, the RSS score increased, whereas the combination of home monitor FEV_1_%pred and RSS in the four weeks to one week before a PEx could discriminate between children who remained stable and children who developed a PEx. Therefore, home monitoring of symptoms and lung function in children with CF is probably useful to detect PEx at an early stage.

## Electronic supplementary material

Supplementary Information
