# Publisher Correction: Early detection of pulmonary exacerbations in children with Cystic Fibrosis by electronic home monitoring of symptoms and lung function

**DOI:** 10.1038/s41598-018-36407-y

**Published:** 2018-12-13

**Authors:** Marieke van Horck, Bjorn Winkens, Geertjan Wesseling, Dillys van Vliet, Kim van de Kant, Sanne Vaassen, Karin de Winter-de Groot, Ilja de Vreede, Quirijn Jöbsis, Edward Dompeling

**Affiliations:** 1 Department of Paediatric Respiratory Medicine, School for Public Health and Primary Health Care (CAPHRI), Maastricht University Medical Centre (MUMC+), Maastricht, The Netherlands; 2 Department of Methodology and Statistics, CAPHRI, MUMC+, Maastricht, The Netherlands; 3 Department of Respiratory Medicine, CAPHRI, MUMC+, Maastricht, The Netherlands; 4 Department of Paediatric Respiratory Medicine, Wilhelmina Children’s Hospital, University Medical Centre Utrecht (UMCU), Utrecht, The Netherlands; 5 Department of Paediatric Respiratory Medicine, Leiden University Medical Centre (LUMC), Leiden, The Netherlands

Correction to: *Scientific Reports* 10.1038/s41598-017-10945-3, published online 27 September 2017

In the original version of this Article, the author Karin de Winter-de Groot was incorrectly indexed. This error has now been corrected.

