# Supplementary material for: Early detection of pulmonary exacerbations in children with Cystic Fibrosis by electronic home monitoring of symptoms and lung function
Source: Sci Rep. 2017 Sep 27;7:12350. doi: 10.1038/s41598-017-10945-3 (PMC5617859; doi:10.1038/s41598-017-10945-3)
Supplement: Supplementary file 1 — Supplementary Information [file 41598_2017_10945_MOESM1_ESM.docx]

**Early detection of pulmonary exacerbations in children with Cystic Fibrosis by electronic home monitoring of symptoms and lung function**

Authors: Marieke van Horck^1^*, Bjorn Winkens^2^_,_ Geertjan Wesseling^3^, Dillys van Vliet^1^, Kim van de Kant^1^, Sanne Vaassen^1^, Karin de Winter-de Groot^4^, Ilja de Vreede^5^, Quirijn Jöbsis^1^, Edward Dompeling^1^

Affiliations:

1 Department of Paediatric Respiratory Medicine, School for Public Health and Primary Health Care (CAPHRI), Maastricht University Medical Centre (MUMC+), Maastricht, The Netherlands

2 Department of Methodology and Statistics, CAPHRI, MUMC+, Maastricht, The Netherlands

3 Department of Respiratory Medicine, CAPHRI, MUMC+, Maastricht, The Netherlands

4 Department of Paediatric Respiratory Medicine, Wilhelmina Children’s Hospital, University Medical Centre Utrecht (UMCU), Utrecht, The Netherlands

5 Department of Paediatric Respiratory Medicine, Leiden University Medical Centre (LUMC), Leiden, The Netherlands

* Corresponding author:

E-mail: Edward.dompeling@mumc.nl

**Supplementary Table S1:**

Definition of pulmonary exacerbation according to the EPIC trial (8).

| **Major criteria** |
| --- |
| Decrease in FEV_1_ >10% from best baseline within past 6 months, unresponsive to beta-2 agonist |
| Oxygen saturation <90% on room air or >5% decline from previous baseline |
| New lobar infiltrate(s) or atelectasi(e)s on chest radiograph |
| Haemoptysis (more than streaks on more than one occasion in past week) |
| **Minor criteria** |
| Increased work of breathing or respiratory rate |
| New or increased adventitial sounds on lung exam |
| Weight loss >5% of body weight or decrease across 1 major percentile in weight percentile for age in past 6 months |
| Increased cough |
| Decreased exercise tolerance or level of activity |
| Increased chest congestion or change in sputum |
